# Epitaxial Metal Electrodeposition Controlled by Graphene Layer Thickness

**DOI:** 10.1021/acsnano.4c02981

**Published:** 2024-05-16

**Authors:** Salem C. Wright, Courtney Brea, Jefferey S. Baxter, Sonakshi Saini, Elif Pınar Alsaç, Sun Geun Yoon, Matthew G. Boebinger, Guoxiang Hu, Matthew T. McDowell

**Affiliations:** † School of Materials Science and Engineering, Georgia Institute of Technology, Atlanta, Georgia 30332, United States; ‡ Department of Chemistry and Biochemistry, Queens College of the City University of New York, New York, New York 11367, United States; § Center for Nanophase Materials Sciences, Oak Ridge National Laboratory, Oak Ridge, Tennessee 37830, United States; ∥ George W. Woodruff School of Mechanical Engineering, Georgia Institute of Technology, Atlanta, Georgia 30332, United States

**Keywords:** electrodeposition, graphene, epitaxy, electrochemistry, EBSD, two-dimensional materials

## Abstract

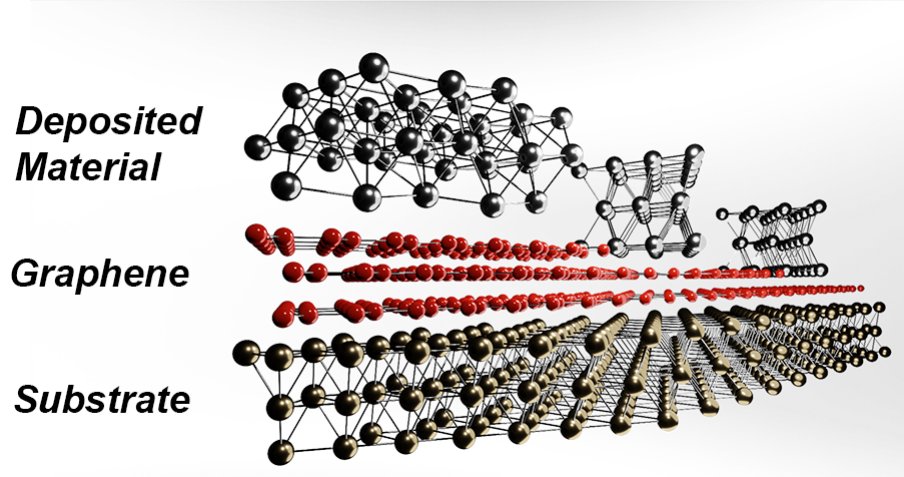

Control over material structure and morphology during electrodeposition is necessary for material synthesis and energy applications. One approach to guide crystallite formation is to take advantage of epitaxy on a current collector to facilitate crystallographic control. Single-layer graphene on metal foils can promote “remote epitaxy” during Cu and Zn electrodeposition, resulting in growth of metal that is crystallographically aligned to the substrate beneath graphene. However, the substrate–graphene–deposit interactions that allow for epitaxial electrodeposition are not well understood. Here, we investigate how different graphene layer thicknesses (monolayer, bilayer, trilayer, and graphite) influence the electrodeposition of Zn and Cu. Scanning transmission electron microscopy and electron backscatter diffraction are leveraged to understand metal morphology and structure, demonstrating that remote epitaxy occurs on mono- and bilayer graphene but not trilayer or thicker. Density functional theory (DFT) simulations reveal the spatial electronic interactions through thin graphene that promote remote epitaxy. This work advances our understanding of electrochemical remote epitaxy and provides strategies for improving control over electrodeposition.

## Introduction

Control over the morphology and structure of metals during electrodeposition is necessary for a variety of applications. For instance, to deposit copper with low sheet resistance for integrated circuits, careful consideration is given to the orientation and microstructure of electroplated copper.^1^ Coatings for corrosion protection also heavily rely on the morphology and structure of the electrodeposited films to achieve desired traits like hydrophobicity and resilience.^2^ Nanostructured electrocatalysts prepared using electrodeposition can take advantage of electrochemical parameters to control surface faceting and thus selectivity and activity.^3^ Additionally, electrodeposition is the mechanism used by battery chemistries with metal anodes.^4^ Because electrodeposition is sensitive to nanoscale surface features, recent work has used 2D materials like graphene at interfaces to control electrodeposition.^5−7^

The electrochemical properties of graphene and other 2D materials have been studied for over a decade,^8^ and early work in this area primarily focused on the role of 2D materials in catalysis;^9^ this has remained an important research area.^10^ The electrochemical differences between basal and edge sites of 2D materials like MoS_2_ were quickly identified, and the inherent anisotropy of 2D materials continues to be a key focus in terms of electrochemical properties.^11−13^ Graphitic nanomaterials like graphene exhibit the same crystallographic anisotropy,^14^ but with the discovery of superconductivity in magic-angle graphene,^15^ tailoring the electrochemical properties of bilayer graphene through direct control of the twist angle between different layers is also of interest. For instance, Yu et al. found that the charge transfer kinetics of bilayer graphene electrodes is highly dependent on the twist angle between the layers.^16^ The electrochemical activity of monolayer graphene has also been enhanced by the introduction of vacancy defects, which modify the electronic structure of graphene and can lower the nucleation overpotential for deposition of materials like Zn.^6,17,18^

One method for utilizing graphene in electrochemical applications is as a substrate for electrodeposition, with some studies suggesting that metals like Zn (which have a low lattice mismatch with graphene) can grow epitaxially on the graphene, while other studies suggest that the Zn orientation depends on the substrate beneath the graphene.^5−7^ However, most work on crystallographic interactions through graphene has used thermal evaporation of semiconductor materials.^19−25^ Because of these conflicting explanations, the precise role that graphene plays as a substrate for deposition remains unclear, and there is the potential for a variety of different forms of epitaxial growth on graphene, including pinhole epitaxy, remote epitaxy, and van der Waals epitaxy.^26,27^ There are numerous benefits to the use of graphene as a substrate for deposition, including its protection against oxidation to maintain a pristine underlying substrate,^28,29^ but the complex interactions of nanoscale sp^2^ materials at electrochemical interfaces suggest that the role of graphene in electrodeposition remains ambiguous.^30,31^

Previous work has found that electrodeposition on monolayer graphene yielded deposits that were epitaxial with the underlying metallic substrate, similar to metal–organic chemical vapor deposition (MOCVD) on graphene-coated semiconductors.^5,19^ Using electron backscatter diffraction (EBSD) and transmission electron microscopy (TEM), Zn and Cu electrodeposited on monolayer graphene were found to grow through “remote epitaxy” in crystallographic alignment with the Cu substrate beneath graphene. While these results support the presence of long-range interactions through graphene, investigating electrodeposition on two or more graphene layers with independent relative rotations would confirm that this phenomenon is not limited to monolayer graphene on Cu grown by CVD, which intrinsically forms with a crystallographic relationship between the graphene and Cu substrate. It has been predicted that electronic interactions through graphene during remote epitaxy of semiconductors will be screened with increasing graphene thickness,^19^ but the effect of graphene layer number has not been considered for electrodeposited metals, which is key for understanding the role that 2D materials play in influencing local electrochemistry and electrochemical characteristics like charge transfer resistance.

Here, we systematically study the influence of the number of graphene layers on the electrochemistry and crystallographic orientation of Zn and Cu electrodeposited from aqueous solutions. The presence of graphene with different layer numbers (mono-, bi-, and trilayer graphene, as well as single crystal graphite) is found to alter electrochemical signatures during electrodeposition. We use electron microscopy to characterize the morphology of Zn and Cu electrodeposits on graphene with different thickness to create a comprehensive understanding of the effects of graphene interlayers on electrochemical growth. The electrochemical results support the presence of remote epitaxy through mono- and bilayer graphene, while this effect is not present when using trilayer or thicker graphene. DFT shows that charge transfer through thin graphene promotes remote epitaxy via alteration of the electronic environment for the first layer of growing Cu. These results reveal the complex electrochemical behavior of thin graphene, with the potential for control over deposited crystallography of various metals through judicious choice of graphene-coated substrate.

## Results and Discussion

**Figure 1:**
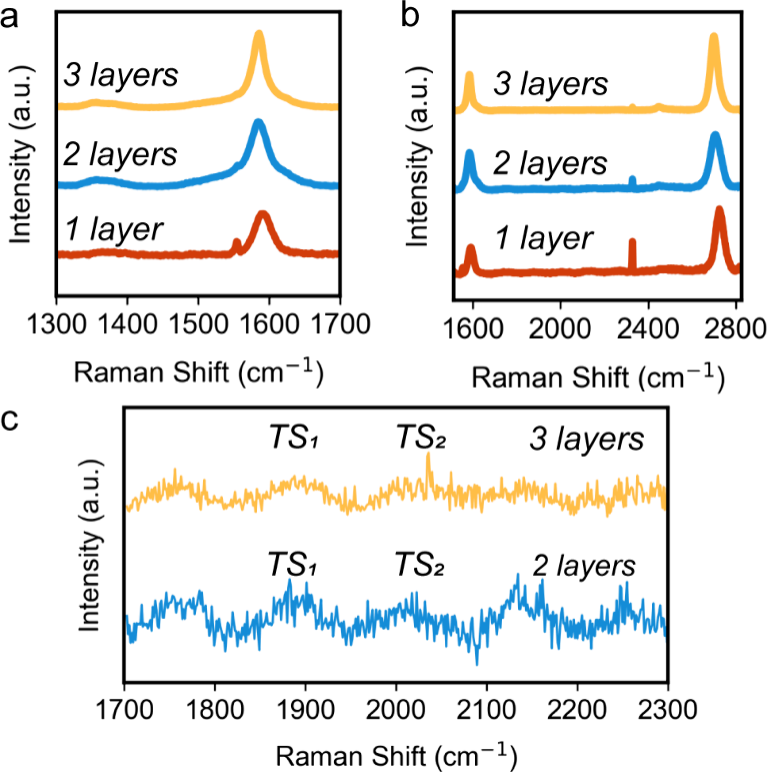
Average Raman spectra (361 acquisitions) taken from a 90 μm^2^ region of as-grown CVD monolayer graphene and bi- and trilayer graphene prepared by polymer-assisted transfer showing (a) the location of the D- and G- peaks and (b) the G- and 2D- peak regions. There is a lack of D-peak intensity near 1350 cm^–1^ in (a), indicating graphene with low defect density. The sharp low intensity peak around 1550 cm^–1^ is an atmospheric O_2_ peak.^40^ The ratio of the 2D peak to G peak intensity in (b) is stable for each graphene thickness, which suggests a lack of Bernal stacking. The sharp peak around 2300 cm^–1^ is an atmospheric N_2_ peak.^40^ (c) Individual Raman spectra taken from bi- and trilayer graphene showing the presence of turbostratic graphene modes labeled TS_1_ and TS_2_.

Monolayer graphene grown on Cu foil by chemical vapor deposition (CVD) was used as an electrode and a substrate for graphene transfer. Bi- and trilayer graphene samples on Cu foils were prepared by transferring the purchased monolayer graphene onto Cu foils that already featured a single layer of CVD-grown graphene. The transfer process was performed once to create bilayer graphene and twice to create trilayer graphene. The transfer process was verified on silicon (Figure S1). Figure 1a shows average Raman spectra from mono-, bi-, and trilayer graphene on the Cu foil. There is little to no D-peak intensity near 1350 cm^–1^ relative to the G peak intensity near 1600 cm^–1^, indicating low defect density in the graphene. The ratios between the G and 2D peaks in Figure 1b are similar for 1-, 2-, and 3-layer graphene. This is inconsistent with AB-stacked graphene, which is the lowest-energy configuration of multilayer graphene.^32,33^ Because our graphene is transferred without intentional alignment, it is thus expected that the graphene samples are randomly rotated relative to each other, which is the signature of turbostratic graphene. Turbostratic graphene can be identified by the presence of low-intensity modes in the 1700–2300 cm^–1^ region of the Raman spectra.^34−37^Figure 1c confirms the presence of the two signature modes of turbostratic graphene, labeled TS_1_ and TS_2_. In addition, there is a low intensity peak around 1750 cm^–1^, typically labeled the *M band*, that is not expected to be present in purely turbostratic graphene, but has previously been found to occur in some turbostratic few-layer graphene samples with relatively low *I*_2D_/*I*_G_ ratio.^38,39^ Therefore, we conclude that we have produced low-defect density bi- and trilayer turbostratic graphene. Together with the low-defect density monolayer graphene grown by CVD, we can therefore use these samples to understand the range of thicknesses at which interactions through graphene are expected to occur while minimizing the influence of defects on the morphology of deposits and their nucleation overpotentials.^19^ By using turbostratic bi- and trilayer graphene, we also minimize the coupling between layers in the samples.

**Figure 2:**
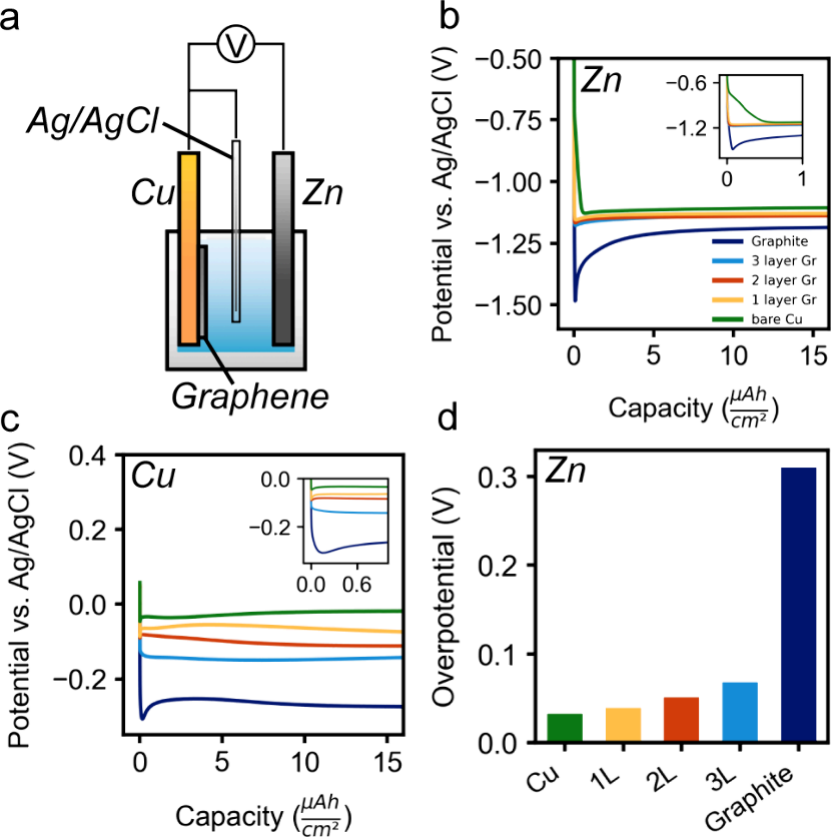
Zn and Cu were electrodeposited in three-electrode cells with Ag/AgCl reference electrodes using a current density of 15 mA cm^–2^ and an areal capacity of 0.17 mAh cm^–2^. Here we show a portion of the areal capacity. (a) Schematic of the electrochemical cell. (b, c) Galvanostatic electrochemical deposition curves of Zn and Cu, respectively, on bare Cu, monolayer graphene, bilayer graphene, trilayer graphene, and graphite. The insets show a smaller capacity range of the same curves. (d) Plot of the nucleation overpotential for different graphene thicknesses for Zn deposition.

Zn and Cu were electrodeposited on bare Cu, mono-, bi-, and trilayer graphene, as well as on bulk graphite single crystals. Three electrode cells were used with Ag/AgCl reference electrodes, Cu/graphene working electrodes, and Cu or Zn metal as the counter electrodes. Zn and Cu were electrodeposited under constant current conditions with a current density of 15 mA cm^–2^ for 40 s (i.e., a capacity of 0.17 mAh cm^–2^). Figure 2a shows a schematic of the experimental cell for these tests. For electrodeposition of Zn (Figure 2b), there is a systematic increase in the nucleation overpotential as the graphene layer thickness increases from single layer to bulk graphite (see Figure 2d). The nucleation overpotential increases from 39 mV for monolayer graphene to approximately 68 mV for trilayer graphene, and it is over 300 mV for graphite. A higher nucleation overpotential for thicker graphene means that there is a shorter time period over which nuclei form, whereas for small nucleation overpotentials nucleation can happen more readily over longer times during deposition.^41^ This suggests that sudden formation of many nuclei is expected for higher nucleation overpotentials. Because of the more complex electrochemical signatures from Cu deposition (Figure S2), classical nucleation and growth analysis was not applied.

For Zn deposited on bare Cu, there is a voltage shoulder (Figure 2b inset), which is expected due to interactions with the native oxide present on Cu. Notably, this shoulder is absent for graphene-coated samples (Figure 2b), as they presumably prevent oxidation of the substrate. This voltage shoulder is also absent for Cu deposition on bare Cu because of the acidic electrolyte used, which dissolves the oxide (Figure 2c inset). The potential of the voltage plateau during growth systematically becomes more negative as Zn is deposited on graphene with increasing layer thickness. This suggests that graphene increases the electrical resistance and/or charge transfer resistance at the interface. To further characterize the impedance of the graphene coatings, we used electrochemical impedance spectroscopy (EIS, Figure S3). For both the Zn and Cu electrolytes in contact with mono-, bi-, and trilayer graphene/Cu substrates, approximate semicircles are observed, with the width of the semicircle steadily increasing with graphene layer number. This finding suggests that the interfacial impedance increases with increasing graphene thickness for both systems. Additionally, the overall impedance of the electrodes in contact with the Cu electrolyte is lower than the Zn electrolyte, which is likely related to differences in charge transfer kinetics between the Zn and Cu species.

**Figure 3:**
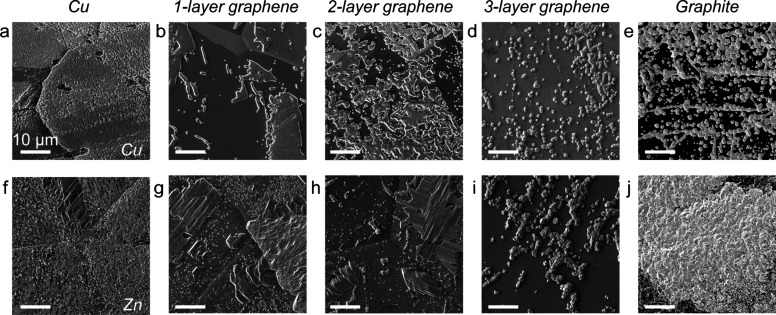
(a–e) SEM images of Cu electrodeposited on (a) bare Cu, (b) monolayer graphene, (c) bilayer graphene, (d) trilayer graphene, and (e) graphite. (f–j) SEM images of Zn electrodeposited on (f) bare Cu, (g) monolayer graphene, (h) bilayer graphene, (i) trilayer graphene, and (j) graphite.

The differences between the Zn and Cu electrochemical curves in Figure 2 are reflected in the morphology of the electrodeposited material observed with SEM, as shown in Figure 3. Figure 3a and 3f show Cu and Zn deposited on bare Cu substrates. In both cases, the deposited metals cover the entire surface of the metal substrates, as observed in previous work.^5^ Cu and Zn electrodeposited on both mono- and bilayer graphene form relatively large, faceted metal deposits that have distinct morphologies on different Cu grains; the underlying grains are distinguishable by the visible grain boundaries that separate them (deposited Cu is shown in Figure 3b, c and deposited Zn is shown in Figure 3g, h). In contrast, Cu and Zn deposited on trilayer graphene exhibit a qualitatively different morphology compared to the material deposited on mono- or bilayer graphene (Figure 3d, i). Specifically, there are many small deposits scattered across the substrate surface. These deposits have the same morphology across the sample surface independent of the underlying Cu grains, in contrast to deposits on mono- and bilayer graphene/Cu, which feature morphology that depends on the underlying Cu grain structure. The deposited Cu on trilayer graphene (Figure 3d) appears to be approximately octahedral in shape, while the Zn deposits (Figure 3i) are boulder-like. Finally, Figure 3e and j show deposited Cu and Zn, respectively, on single crystal graphite. The deposits show similar morphology to the deposits on trilayer graphene, but they cover more of the graphite surface. Cross-sectional cryogenic focused ion beam (FIB) SEM imaging was used to further investigate the morphology of the Cu deposits on mono-, bi-, and trilayer graphene (Figure S4). From these data, the thicknesses of the Cu deposits are similar on the electrodes with different graphene layer numbers.

The morphology of the metal deposited on mono- and bilayer graphene is distinct from the deposits on thicker graphene because for the mono and bilayer graphene, the underlying Cu can exert an influence on the structure of the deposits.^5^ The results in Figure 3 show that this interaction dissipates for three or more graphene layers, presumably because the thicker graphene limits the electronic interaction between substrate and deposit.^19^ The similar deposit morphologies on trilayer graphene and bulk graphite suggest similar interfacial interactions during nucleation and growth. Furthermore, the morphologies in Figure 3 are consistent with the nucleation overpotentials in Figure 2. Increasing nucleation overpotentials with thicker graphene toward graphite result in more nuclei that form. Finally, the bare Cu and Zn samples are covered with material likely because of the heterogeneous/defective nature of the surfaces.

**Figure 4:**
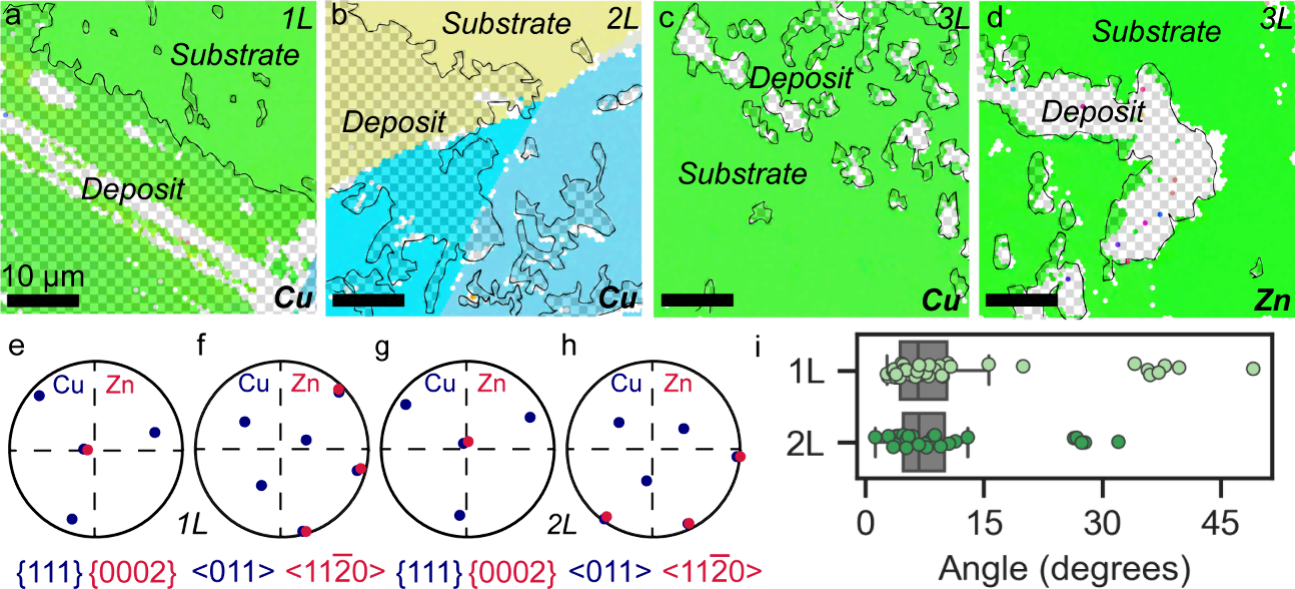
(a, b) Electron-backscatter diffraction inverse pole figure maps of the orientation of deposited Cu on (a) monolayer and (b) bilayer graphene. The regions of deposited Cu are outlined in black and are shaded with a halftone overlay. The Cu deposits have the same orientation as the grains on which they are deposited, as represented by the same colors in the map. (c, d) EBSD inverse pole figure maps of the orientation of (c) deposited Cu and (d) Zn on trilayer graphene. The regions of deposited Cu and Zn are outlined in black but are not able to be indexed, suggesting very small grain size. (e, f) stereographic projections of Zn and Cu close-packed planes (e) and directions (f) from deposited Zn on a single Cu grain on monolayer graphene. (g, h) stereographic projections of Zn and Cu close-packed planes (g) and directions (h) from deposited Zn on a single Cu grain on bilayer graphene. (i) Boxplot of the angles between the close-packed Zn and Cu planes for a larger number of deposited Zn crystals on mono- and bilayer graphene.

We quantitatively investigated the crystallographic orientation relationships between the Cu or Zn deposits and the underlying Cu substrate using electron backscatter diffraction (EBSD). Figure 4a and b show inverse pole figure maps of deposited Cu on mono- and bilayer graphene, respectively. The colors on the maps, which represent crystallographic orientation, are the same for the deposited Cu and the underlying Cu substrate, indicating that they are the same orientation. For instance, Figure 4b shows three different underlying Cu grains with different colors, and the Cu deposits (shown outlined with halftone overlay) exhibit identical colors. For trilayer graphene, however, EBSD is unable to index the orientation of the deposited material likely because of the small crystallite size of the deposits (Figure 4c, d). This suggests a lack of crystalline registry. Figure 4e and 4f show stereographic projection analysis from Zn deposited on monolayer graphene covering a single Cu grain. The close-packed {0002}_Zn_ pole (i.e., the basal plane of the Zn hexagonal structure) overlaps with one of the {111}_Cu_ poles, indicating directional alignment (Figure 4e). Full crystallographic alignment is further confirmed by the alignment of the Zn close-packed directions and Cu close-packed directions from the same crystal (Figure 4f). Figure 4g and 4h are an example of the same behavior for deposited Zn on bilayer graphene on one underlying Cu grain. The stereographic projection of the red Zn poles closely overlaps with the blue poles of the underlying copper in both close-packed plane orientation (Figure 4g) and close-packed direction (Figure 4h). Stereographic projection analysis is less illustrative for Cu deposition on mono- and bilayer graphene/Cu substrates because the deposit orientation is not discernible from the underlying Cu orientation (as shown in Figure 4a, b). Overall, these data suggest that the electrodeposition of Cu and Zn on both mono- and bilayer graphene results in full crystallographic alignment with the underlying Cu substrate (i.e., remote epitaxy).

The crystallographic orientation relationship between the deposited Zn and the underlying Cu substrate on mono- and bilayer graphene is further confirmed by quantifying the angle between the close-packed Zn and Cu planes from many individual grains, with results shown in Figure 4i. The median angle between the Zn and Cu plane normals is 6.7° for monolayer graphene and 6.8° for bilayer graphene, with the majority of the grains clustered around these values. The slight offset is likely due to the {111} faceted surface of the Cu grains, which are primarily {111} and {001} in orientation.^42^ The results in Figure 4i demonstrate the consistent crystallographic alignment for mono- and bilayer graphene/Cu over many individual crystals.

**Figure 5:**
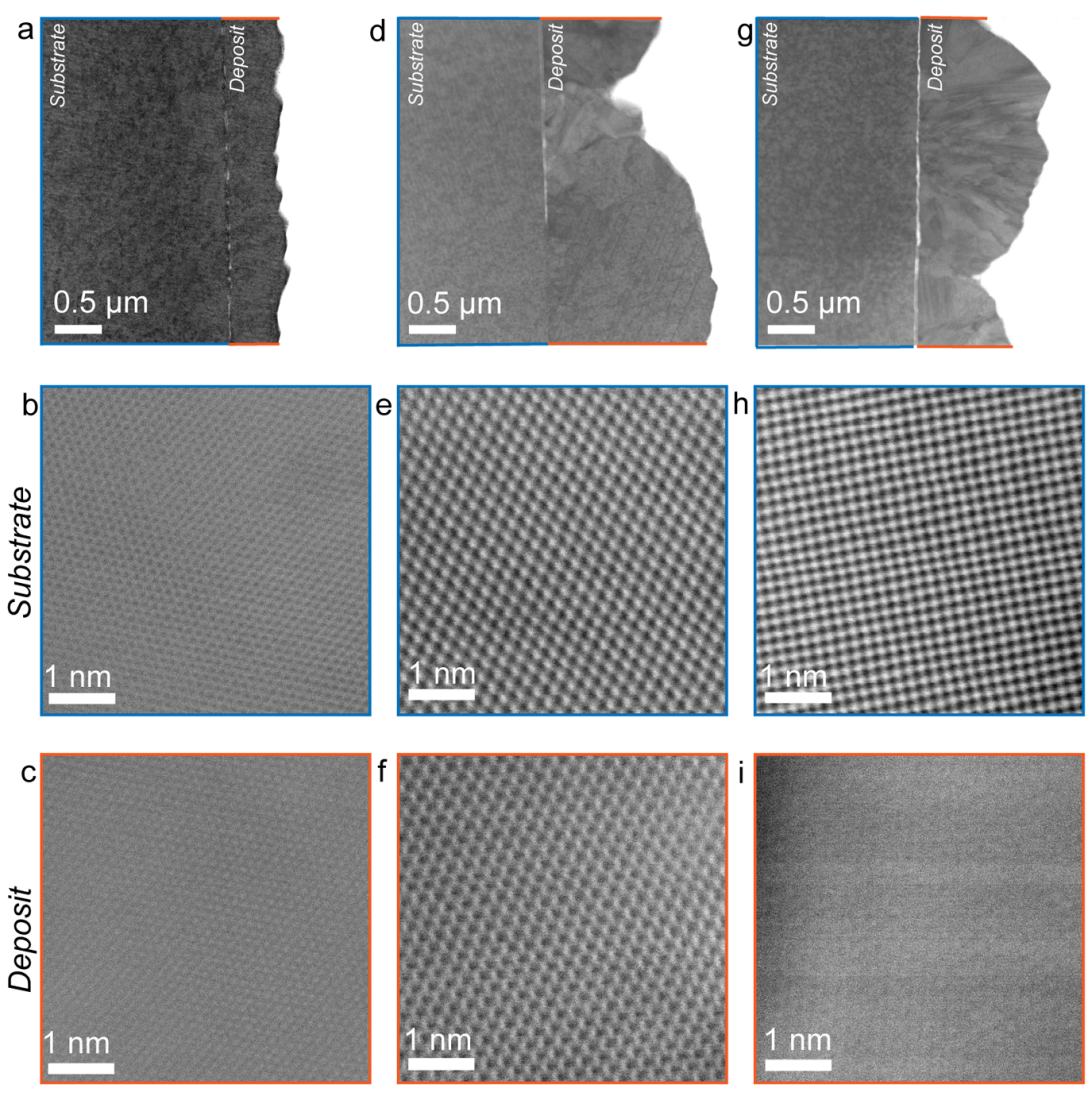
(a, d, g) ABF STEM images of cross-sectioned lamella of Cu deposited on mono- (a), bi- (d), and trilayer (g) graphene. (b, c) ADF STEM images of the substrate (b) and deposit (c) for a sample with Cu grown on monolayer graphene/Cu. (e, f) ADF STEM images of the substrate (e) and deposit (f) for a sample with Cu grown on bilayer graphene/Cu. (h, i) ADF STEM images of the substrate (h) and deposit (i) for a sample with Cu grown on trilayer graphene/Cu.

We also carried out scanning transmission electron microscopy (STEM) to investigate the nature of the interface between the electrodeposits and the substrate. Figure 5a, d, g show annular bright-field (ABF) STEM cross sectional images of Cu deposited on mono-, bi-, and trilayer graphene, respectively. The Cu deposited on monolayer graphene is highly uniform and thinner than the Cu deposited on bi- and trilayer graphene. For mono- and bilayer graphene, there is noncontinuous lighter contrast at the interface, while the trilayer graphene sample shows a continuous lighter layer at the interface (Figure 5i). This brighter contrast at the interface is due to the presence of graphene of different thicknesses.

Figure 5b–c, e–f, and h–i show separate high-magnification annular dark-field (ADF) STEM images of the substrates and the deposits grown on those substrates for the three graphene layer thicknesses. For monolayer (Figure 5b, c) and bilayer (Figure 5e, f) graphene, the deposited material and the substrate lattices show identical crystallographic orientation along the [1̅11] zone axis for the monolayer sample and along the [011] zone axis for the bilayer sample. The Cu deposited on bilayer graphene does show stacking faults, with more defects than the sample grown on monolayer graphene (Figure S5). Figure 5h, i show high-magnification images of the substrate and deposited material when trilayer graphene is present. The lattices are clearly not aligned in this case. The substrate is aligned along the [001] zone axis, while the region of the deposit shown is aligned along the [011] zone axis.

**Figure 6:**
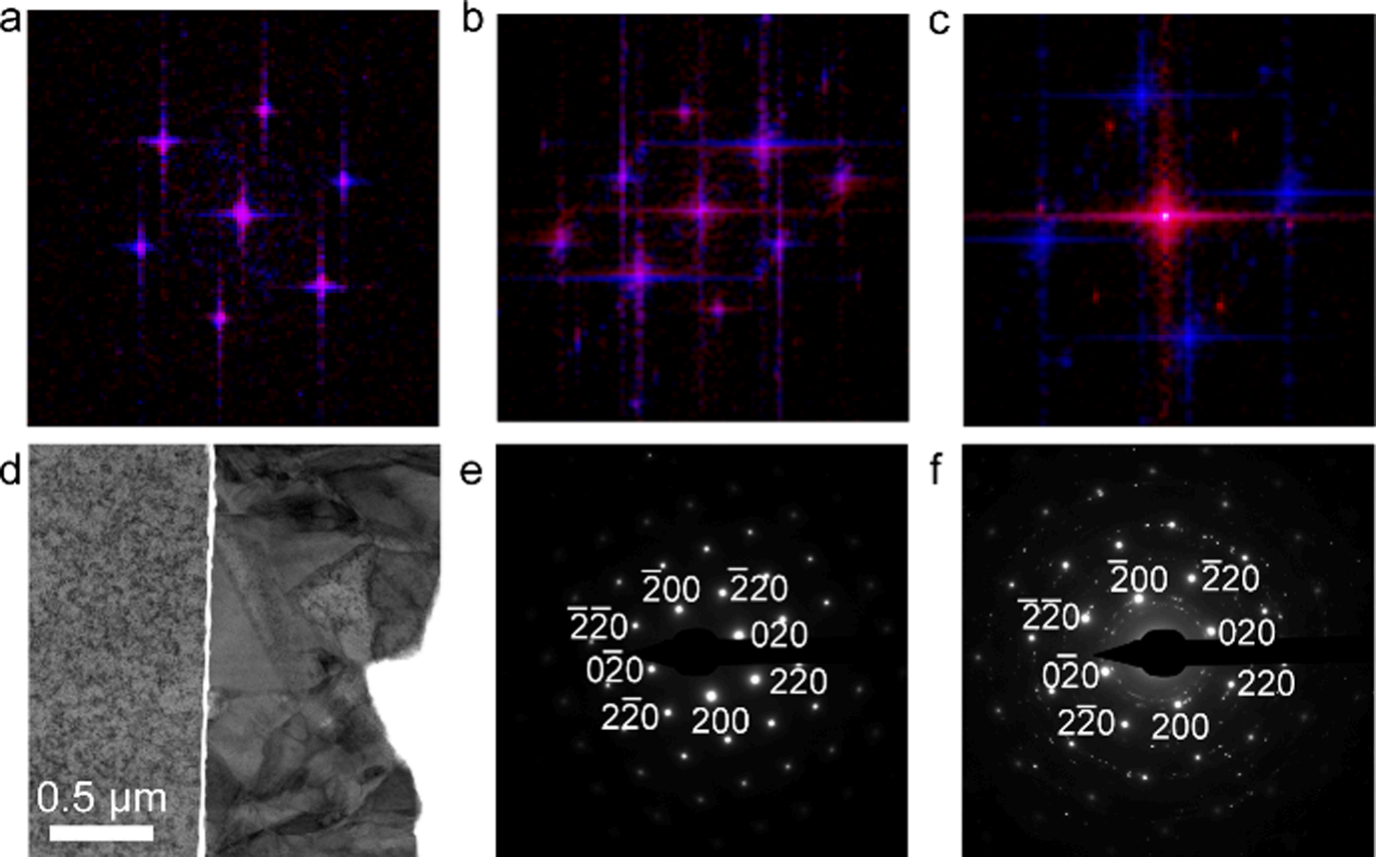
(a–c) Overlaid FFTs of the Cu substrate and Cu deposit with monolayer (a), bilayer (b) and trilayer (c) graphene. The substrate FFT is shown in blue and the deposit FFT is shown in red; thus, overlapping areas are purple. (d–f) ABF STEM image of Cu deposited on a trilayer graphene/Cu (substrate is the left portion of the image). (e) SAED pattern of the Cu substrate showing a single Cu crystal indexed to the [001] zone axis. (f) SAED pattern of both the Cu substrate and the Cu deposit showing the single crystal pattern of the substrate and the polycrystalline patter of the deposit.

Further confirmation of registry is provided by fast Fourier transform (FFT) analysis. Figure 6a shows FFTs of the two lattice images from Figure 5b, c (monolayer graphene), demonstrating complete overlap. Figure 6b shows similar results for bilayer graphene. For Cu deposited on trilayer graphene, the substrate and deposit do not have the same orientation, as evidenced by the differences in the overlaid FFTs (Figure 6c). Further STEM imaging of the trilayer sample (Figure 6d) revealed that the deposited Cu exhibited significant contrast variations due to polycrystallinity on the micron scale and crystal defects, in contrast to the relatively large grains in the Cu substrate. The selected area electron diffraction (SAED) of the Cu substrate (Figure 6e) shows a single crystal Cu pattern, while the SAED pattern of both the Cu substrate and Cu deposit (Figure 6f) for the trilayer graphene sample confirms the randomly oriented polycrystalline nature of the deposit. This differs from the mono- and bilayer samples, which feature deposits with much larger crystal size that are crystallographically aligned with the substrate through the graphene. Taken together, these imaging and diffraction data confirm the local registry between the substrate and deposit across mono- and bilayer graphene via remote epitaxy, while there is no crystallographic registry between the substrate and deposit for the three-layer graphene sample. Thus, we conclude that structural alignment is lost with three or more graphene layers.

**Figure 7:**
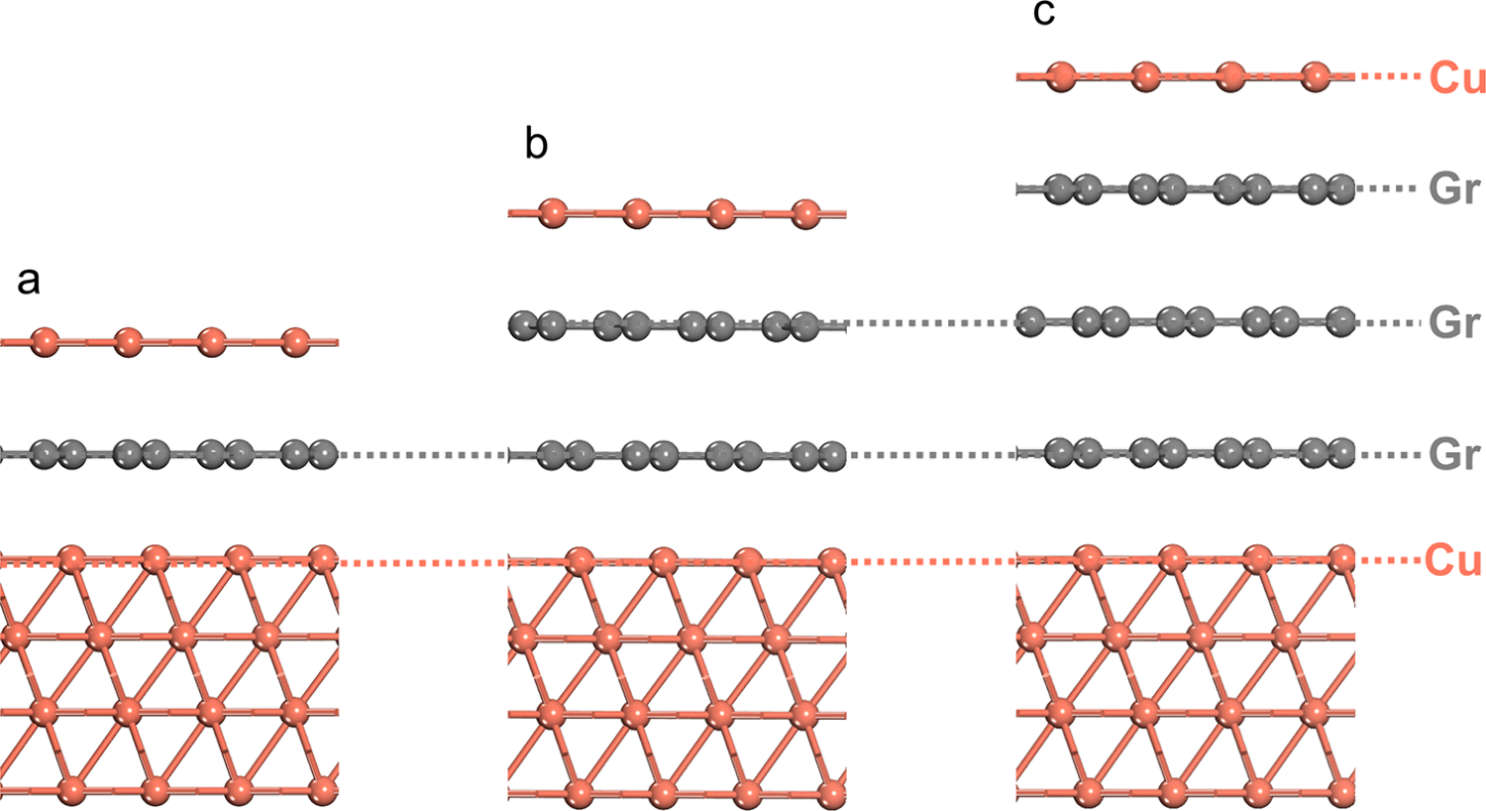
Atomic models for DFT calculations of Cu deposition on (a) monolayer, (b) bilayer, and (c) trilayer graphene/Cu(111).

We performed DFT calculations to understand how the thickness of graphene layers affects the interaction between the deposited Cu and the underlying Cu substrate. Figure 7 shows the atomic models used, where different numbers of graphene layers were inserted between a Cu monolayer and a Cu(111) substrate. After geometry optimization, the distance between the Cu monolayer and the top graphene layer was found to increase from 3.202 Å for monolayer graphene to 3.254 Å for trilayer graphene. We then calculated the energy required to exfoliate the Cu monolayer away from the graphene/Cu(111) substrate, which was found to be 208 meV/Cu, 194 meV/Cu, and 189 meV/Cu for monolayer, bilayer, and trilayer graphene, respectively. These calculations demonstrate that as the graphene thickness increases from monolayer to trilayer, the interaction energy between the Cu deposit and substrate becomes weaker.

**Figure 8:**
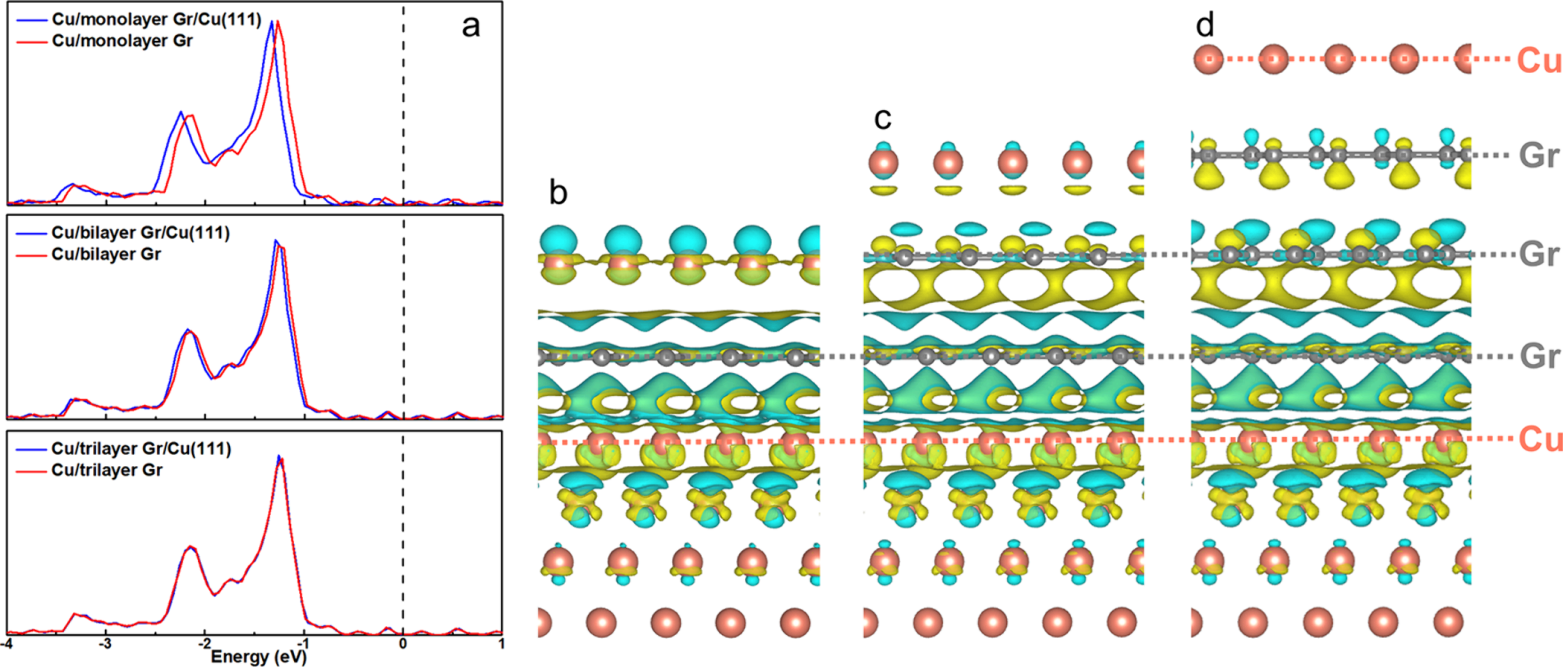
(a) Local electronic density of states of the deposited Cu on graphene (red) and graphene/Cu(111) substrate (blue). The top, middle, and bottom panels show mono-, bi-, and trilayer graphene. The Fermi level is set as zero. (b–d) Isosurface plots of the charge density difference for Cu deposition on (b) monolayer, (c) bilayer, and (d) trilayer graphene/Cu(111) substrate. The yellow color indicates charge accumulation, and the cyan color indicates charge depletion. The value of the isosurface is set as 0.0006 eV/Å^3^.

To further understand the electronic interaction between the Cu deposit and the substrate through graphene, we plotted the local electronic density of states of the Cu deposit on graphene and graphene/Cu(111). As shown in Figure 8a, a negative shift of the local density of states was observed when the Cu(111) substrate was included. This effect is evident for monolayer and bilayer graphene, but it disappears for trilayer graphene, corresponding to a negligible electronic interaction between the Cu deposit and the substrate for trilayer graphene. This is in good agreement with the experimentally observed remote epitaxy occurring on monolayer and bilayer graphene but not trilayer or thicker graphene, and it can further be explained by the presence of charge transfer from the Cu substrate to the deposit through the thinner graphene. Figure 8b–d shows the charge density difference plots. Electronic charge can be transferred from the Cu substrate to the deposit through monolayer or bilayer graphene. Specifically, Bader charge analysis revealed a charge transfer of 0.152 |*e*| for monolayer graphene and 0.068 |*e*| for bilayer graphene. However, for trilayer or thicker graphene, the charge transfer is inhibited, explaining the negligible electronic interaction between the substrate and deposit. As a result, remote epitaxy is not expected to occur in this case.

## Conclusions

In conclusion, we have shown that electronic interactions from an underlying Cu substrate through mono- and bilayer graphene facilitate epitaxial growth of electrodeposited Zn and Cu. STEM imaging confirms the agreement in orientation between substrate and deposit near their interface for the mono- and bilayer samples, while the substrate and deposit are not aligned in the case of deposition on trilayer graphene. Analysis of the alignment of close-packed planes and directions in the substrate and deposit from EBSD data further confirms epitaxial electrodeposition for mono- and bilayer graphene samples. DFT calculations reveal electronic interactions between the substrate and deposit through 1- and 2-layer graphene, but inhibited charge transfer through 3-layer graphene. The understanding herein that graphene with multiple layer thicknesses can facilitate crystallographic interactions between underlying metal and electrodeposited material has implications for control of the electrochemical growth processes of metals. Since graphene can prevent substrate oxidation and also be transferred to a wide variety of substrates, these findings suggest that a wide variety of substrate materials can be selected to influence the deposition of different deposited metals. Selection of substrates with appropriate crystallographic relationships may enable controlled growth of metals for an array of applications ranging from catalysis to surface coatings.

## Methods

### Raman Spectroscopy

Raman spectroscopy and mapping were performed in the Renishaw Raman Spectrometer-Vis/near-IR with a 65 mW 488 nm laser. Raman maps comprised of 361 individual spectra over 90 μm^2^ regions were collected at 50% laser power with an exposure time of 1 s for all samples except for the 1L graphene sample spectra in Figure 1b, which was collected with a 5 s exposure time to increase counts. A noise filter was applied to the spectra from the maps and the background subtracted before being averaged. The spectra in Figure 1c were collected at 100% laser power with an exposure time of 240 s, after which the background was subtracted from their spectra. In all cases, a 50× objective was used with a 2400 I/mm grating.

### Graphene Transfer

CVD monolayer graphene on one side of Cu foil was purchased from General Graphene. PMMA (495 A4 Kayaku) was spin coated at 1000 rpm for 60 s on the graphene side of the monolayer graphene/Cu foil followed by a cure for 3 min at 100 °C. The backside Cu was then etched in 0.1 M ammonium persulfate solution. After etching overnight, the remaining PMMA/graphene was transferred to four separate DI water baths for cleaning before transferring to the substrate of choice. After transferring, the sample was heated for 15 min at 150 °C. To remove PMMA residue, the sample was left in acetone overnight. Finally, the resulting sample was washed in IPA for 30 min and then washed in DI water for 15 min. To create bilayer graphene, monolayer graphene was transferred to the purchased CVD monolayer graphene on Cu. To create trilayer graphene, monolayer graphene was transferred to bilayer graphene created by PMMA transfer.

### Electrodeposition and Electrochemical Impedance Spectroscopy

Electrodeposition was carried out in three-electrode cells using Ag/AgCl in 1 M KCl reference electrodes purchased from CH Instruments. Zn was electrodeposited by constant current with a current density of 15 mA cm^–2^ and a capacity of 0.17 mAh cm^–2^. 2.0 M ZnSO_4_ (Sigma-Aldrich) was used as the electrolyte for Zn deposition with Zn metal (Sigma-Aldrich) as the counter electrode. Cu was electrodeposited by constant current with a current density of 15 mA cm^–2^ and a capacity of 0.17 mAh cm^–2^. Twenty g of CuSO_4_–5H_2_O (Sigma-Aldrich) was dissolved in 200 mL of 0.5 M H_2_SO_4_ electrolyte (Supelco) and used as the electrolyte for Cu deposition with Cu metal (MTI) as the counter electrode. The Cu metal used as a counter electrode and substrate deposition was prepared by heating to 1020 °C in a flowing forming gas environment (Ar 95% H_2_ 5%) for 1 h. Kish graphite (2Dsemiconductors) was also used as a substrate for Zn and Cu electrodeposition. EIS was carried out using a Bio-Logic potentiostat with frequencies from 1 MHz to 1 Hz with a 30 mV voltage perturbation for various graphene/Cu electrodes with Zn or Cu counter electrodes in their respective electrolytes. Extraneous impedance values were truncated at high and low frequencies for the Cu sample.

### Scanning Electron Microscopy and FIB-SEM

SEM images were collected at 5 kV using a Tescan Mira3 XM FEG-SEM. Cryogenic FIB cross sectioning of deposited Cu on monolayer, bilayer, and trilayer graphene/Cu was performed using a Thermo Fisher Helios 5 CX dual beam FIB with a milling current of 0.77 nA and a polishing current of 0.4 nA at −140 °C.

### Electron Backscatter Diffraction

After mounting foils on a 70° holder with no surface milling, scans were collected at 20 kV using the EDAX/AMETEK electron backscatter detector and filtered to only include data with a confidence index of 0.1 or higher for analysis in the Matlab package MTeX.^43^ Crystallographic models were generated using *CrystalMaker* from CrystalMaker Software Ltd. (www.crystalmaker.com).

### Scanning Transmission Electron Microscopy and Transmission Electron Microscopy

Aberration-corrected scanning transmission electron microscopy (STEM) images and structural measurements of the FIB-prepared lamellae were collected on a JEOL NEOARM at Oak Ridge National Laboratory at 200 kV. For the selected area electron diffraction experiments an FEI Titan aberration-corrected transmission electron microscope was used at 300 kV.

### Focused Ion Beam Sample Preparation

Samples were prepared by first depositing a conductive protective carbon layer using a sharpie marker dot, before depositing ion beam carbon and tungsten on the Cu-graphene samples. A Hitachi NB5000 FIB instrument was then used to prepare lamellae for transmission electron microscopy (TEM) characterization following established protocols, with a 40 kV beam used to thin down the lamellae to ∼300 nm and then a 10 kV beam to thin down to the final electron beam transparent thickness.^44^

### Density Functional Theory

DFT calculations were performed using the Vienna ab initio simulations package (VASP).^45,46^ The electron exchange-correlation was represented by the functional of Perdew, Burke and Ernzerhof (PBE) of generalized gradient approximation (GGA).^47^ The ion–electron interaction was described with the projector augmented wave (PAW) method.^48^ A cutoff energy of 400 eV was used for the plane-wave basis set. The energies were converged with a 1 × 10^–4^ eV tolerance, and the forces were optimized to within 0.03 eV/Å.

Cu/Gr/Cu(111) systems were modeled by different numbers of graphene layers inserted between a Cu monolayer and four layers of Cu(111) in (4 × 4) lateral cells. During geometry optimization, the Cu monolayer, graphene layers, and top two layers of Cu(111) were allowed to move. A vacuum of 20 Å along the *z*-direction was used. A (3 × 3 × 1) Monkhorst–Pack k-point mesh was used to sample the Brillouin zone. Bader analysis was used to calculate the partial atomic charges.^49^ The DFT-D3 method was used to include the van der Waals interactions.^50^

## Supporting Information Available

Raman spectroscopy of one- and two-layer graphene prepared by PMMA transfer to silicon; electrochemical deposition data for Cu deposition on monolayer graphene; electrochemical impedance spectroscopy of one-, two-, and three-layer graphene in contact with Zn and Cu electrolytes; cross-sectional SEM images of Cu deposition on one-, two-, and three-layer graphene prepared with cryogenic FIB; ADF STEM image of Cu deposited on bilayer graphene showing stacking faults (PDF)
