# Supplementary material for: Epitaxial Metal Electrodeposition Controlled by Graphene Layer Thickness
Source: ACS Nano. 2024 May 16;18(21):13866–75. doi: 10.1021/acsnano.4c02981 (PMC11140832; doi:10.1021/acsnano.4c02981)
Supplement: Supplementary file 1 — nn4c02981_si_001.pdf [file nn4c02981_si_001.pdf]

Supporting Information

Epitaxial Metal Electrodeposition Controlled by Graphene Layer Thickness

Salem C. Wright[a], Courtney Brea[b], Jefferey S. Baxter[c], Sonakshi Saini[a], Elif Pınar Alsaç [d], Sun Geun Yoon[d], Matthew G. Boebinger[c], Guoxiang Hu[a], Matthew T. McDowell[ad]*

[a]School of Materials Science and Engineering, Georgia Institute of Technology, Atlanta, Georgia 30332, United States

[b]Department of Chemistry and Biochemistry, Queens College of the City University of New York, New York, New York 11367, United States

[c]Center for Nanophase Materials Sciences, Oak Ridge National Laboratory, Oak Ridge, Tennessee 37830, United States

[d]George W. Woodruff School of Mechanical Engineering, Georgia Institute of Technology, Atlanta, Georgia 30332, United States

*E-mail: mattmcdowell@gatech.edu

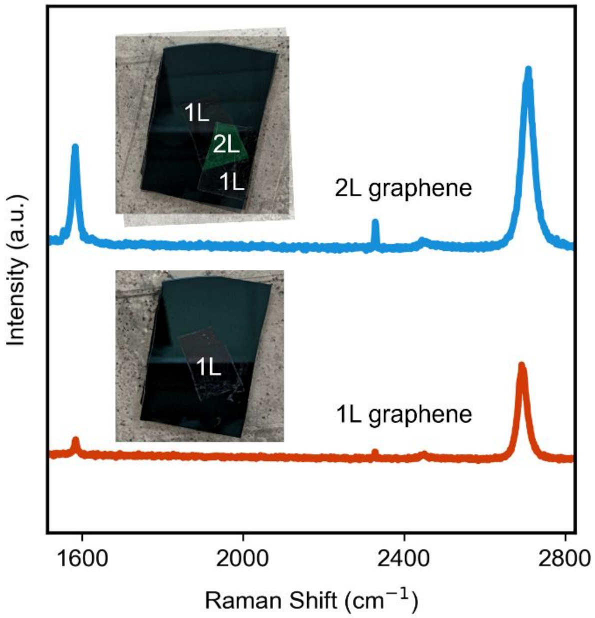

Figure S1: Raman spectra taken from graphene transferred to Si after one transfer (1L) and after transferring a second graphene layer (2L). Optical images are shown after transfer prior to PMMA removal with acetone. The optical image showing the 2L region is the composite of both individual 1L images after transfer prior to acetone treatment.

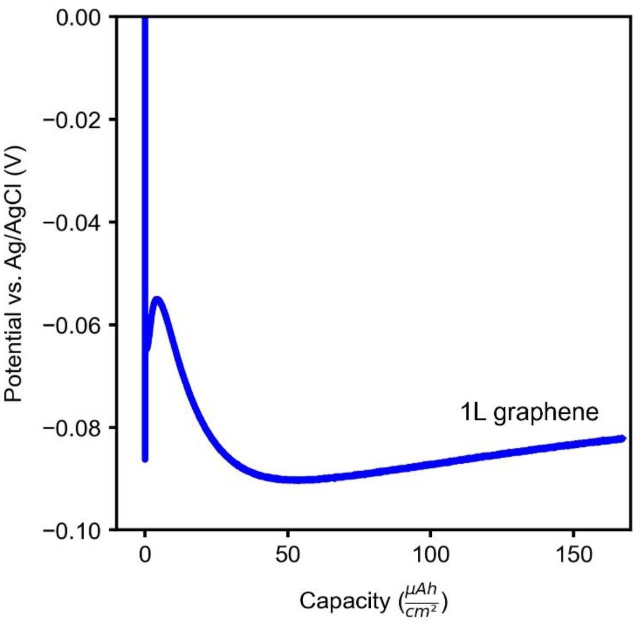

Figure S2: Electrochemical deposition curve for Cu deposition on monolayer graphene at a current density of 15 mA cm^{-2} and an areal capacity of 0.17 mAh cm^{-2}.

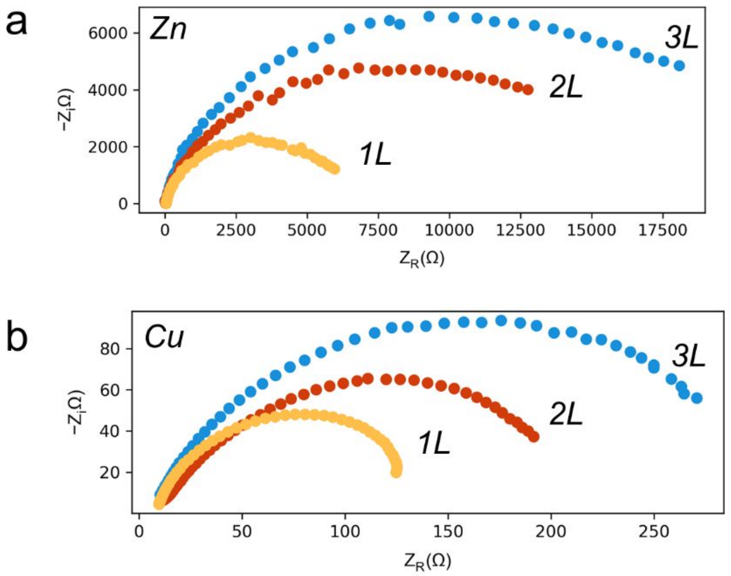

Figure S3: Electrochemical impedance spectroscopy of monolayer (1L), bilayer (2L), and trilayer (3L) graphene/Cu electrodes in contact with Zn (a) and Cu (b) electrolytes.

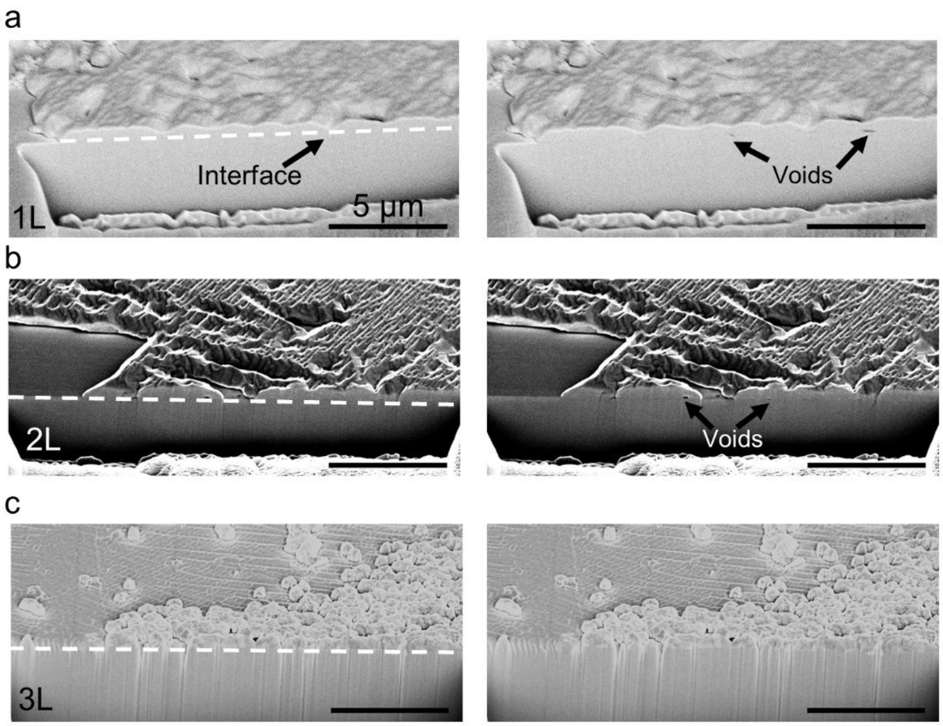

Figure S4: Cross-sectional SEM images of Cu deposited at 15 mA cm^{-2} for 40 s on (a) monolayer (1L), (b) bilayer (2L), and (c) trilayer (3L) graphene/Cu. All scale bars are 5 μm. Cross-sectioning was done using cryogenic FIB. The images in the left column have dotted lines denoting the graphene interface, while the images in the right column are the same images but without the lines.

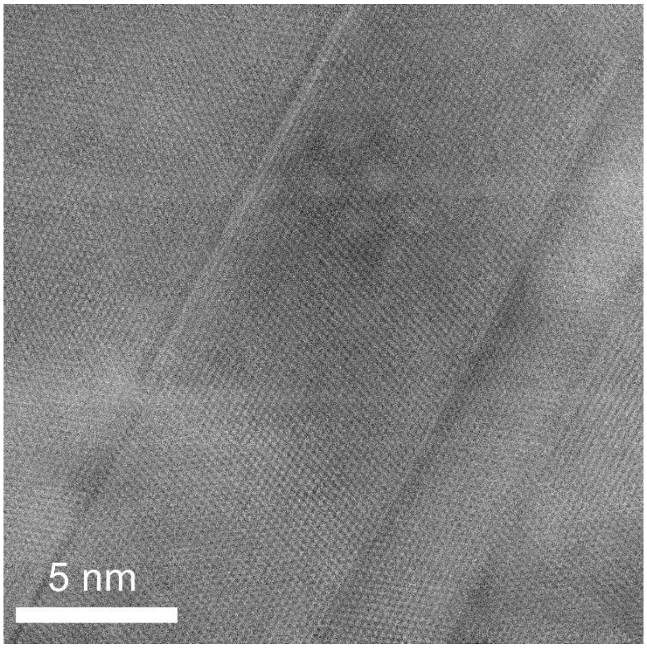

Figure S5: ADF STEM image of Cu deposited on bilayer graphene showing stacking faults.
